# A Critical Assessment of the Peer Review Process in *Life*: From Submission to Final Decision

**DOI:** 10.3390/life13071603

**Published:** 2023-07-21

**Authors:** Pabulo Henrique Rampelotto

**Affiliations:** 1 Bioinformatics and Biostatistics Core Facility, Institute of Basic Health Sciences, Federal University of Rio Grande do Sul, Porto Alegre 91501-970, Brazil; prampelotto@hcpa.edu.br; 2 Graduate Program in Biological Sciences: Pharmacology and Therapeutics, Federal University of Rio Grande do Sul, Porto Alegre 91501-970, Brazil

## 1. Towards a Transparent, Fair, and Reliable Peer Review Process

In the world of academic research and scientific publishing, the process of peer review plays a pivotal role. It has been an integral part of the scientific community for over 300 years as it serves as an essential mechanism for ensuring the novelty, quality, and integrity of research before it is published in reputable journals. However, the traditional model of peer review has remained largely unchanged. This conventional approach relies on undisclosed evaluations, keeping the entire process veiled from the scientific community. Unfortunately, the existing system lacks adequate transparency and quality, leading to persistent concerns regarding potential biases and misconduct practices.

One of the most challenging tasks for authors is to find clear and reliable data on a journal’s performance, especially related to how their manuscripts are handled after submission and the usual time taken to review their work. This transparency is essential for authors, so that they may understand how their work is being evaluated, and can help readers assess the quality of the journal and the published articles. Even nowadays, most researchers gather such information via word-of-mouth or by accessing blog forums, where other authors often share their personal experiences related to journals.

At *Life*, we are committed to providing a transparent, fair, and reliable peer review process. While detailed information can be found on our website [1], I would like to personally highlight some crucial additional details for our authors and readers. By doing so, my aim is to help authors and readers make better-informed decisions regarding where to publish their work, while also enhancing trust and recognition for the tremendous work of our editors and reviewers.

## 2. Visual Metrics and Open Science Practices

Visual metrics on journal websites can increase trust in the peer review system by providing a more transparent and accessible view of the peer review process. Such metrics can include information pertaining to the number of reviewers involved in the process, the time taken for review, and the acceptance rate of papers. This transparency can help authors to understand how their work is being evaluated and can help readers to assess the quality of published research.

While it is quite difficult for authors and readers to find data on a journal’s performance (publishers often keep this information locked), MDPI adopts open science practices by providing a range of tools and resources on the website of each journal, including information measuring a journal’s impact, as well as article-level metrics that can help authors to measure the impact of their research. Each metric can place different emphasis depending on the data source, calculation method, or usage context. To ensure the responsible utilization of research metrics, MDPI offers a variety of statistically sound metrics that provide authors with valuable information in order to assist them in their selection. The key metrics are found right at the top of the journal’s main page [2], including its indexing in major databases (with a direct link to the journal in each database), its Impact Factor and CiteScore, the journal’s rank in JCR and CiteScore, and the length of the peer review process. However, the complete array of metrics and statistics can be found at https://www.mdpi.com/journal/life/stats [3]. The objective is clear: to make these metrics transparent to any readers and authors.

In addition to the mentioned metrics, we offer some unique features that were implemented nearly 10 years ago, like the inclusion of authors’ testimonials [4,5]. These testimonials provide insights into the entire editorial journey, including its quality and the time it has taken. By sharing the experiences of those who have closely collaborated with us, we highlight the high quality and effectiveness of our work.

## 3. The Efficient Review Process

While the journal adopts a rigorous and transparent peer review process, the mean time from the manuscript’s submission to the first decision is relatively fast when compared to most journals (approximately 16.9 days after submission, median values for papers published in this journal in the first half of 2023). Even major open-access journals are known to experience lengthy delays in the peer review, with 2 to 6 months as the standard. While this may sound contradictory (so far, the consensus is that a qualified peer review should take several months), it is easy to understand the effectiveness of the peer review process in *Life*. Journals often overcomplicate the peer review process, either due to overly complex processes or the inability to find suitable reviewers. MDPI, on the other hand, has developed a refined system that minimizes these issues.

First, the submission system is designed to enhance the authors’ experience, providing a user-friendly, efficient, and secure platform for submitting articles. The instructions offer clear guidelines, ensuring that users know exactly what is required of them, including the permissions due when necessary, and the submission system is efficient and streamlined, allowing authors to submit their documents swiftly and easily, which minimizes the time and effort required for submission [6]. The journal’s template (available in Microsoft Word and LaTeX formats) also provides an exact view of the final product and a sense of what to expect from the manuscript if it is accepted, which also facilitates the peer review process for the editorial board and reviewers [6].

The inability to find suitable reviewers is a major issue for the publishing industry, mainly due to the lack of due recognition associated with the increasing demand for reviewers. Reviewers typically do not receive recognition or monetary compensation for their efforts, making engaging in this task less appealing to researchers. This lack of recognition creates a disincentive for potential reviewers to commit their time and expertise to the reviewing process. Additionally, as the number of peer-reviewed journals and submitted articles continues to expand, the demand for reviewers also increases. This surge in demand, coupled with the reluctance of researchers to take on reviewing responsibilities (especially without any compensation), creates a shortage of available reviewers.

Publishers, on the other hand, are reluctant to provide any monetary compensation to reviewers due to the major financial burden it may exert on the company. Paying each reviewer for their contribution seems unfeasible, especially when several reviewers are assigned to an article (in *Life*, it is common to have 3–5 reviewers per article). The truth is that providing reviewers with payment would only lead publishers to raise their prices for subscription or open access. To overcome this problem, MDPI provides vouchers entitling reviewers to a discount on the Article Processing Charges of their next publication in any MDPI journal. It is a fair and clever solution that demonstrates our appreciation for the work performed by reviewers, and does not have a direct impact on the publisher’s revenue.

The reviewers also receive a personalized reviewer certificate for each reviewed manuscript and are eligible to be considered for the annual “Outstanding Reviewer Awards” [7]. In addition, reviewers may create a profile on Web of Science Reviewer Recognition Service (formerly Publons) and have their reviewing activity automatically added for participating journals [8]. Profiles on the Web of Science Reviewer Recognition Service can also be integrated with ORCID.

Another interesting feature provided by MDPI is the Reviewer Article Selector, a tool that is available for board members and volunteer reviewers. Using this function, reviewers can select manuscripts that spark their interest and apply to review them at their own convenience. By allowing reviewers to choose when they are available and review manuscripts that are best suited to their research interests and expertise, we can continue to provide authors with a robust and rapid peer review process. To maintain the high standards of our peer review process, once the application is received, the editorial office of the journal will check for any existing conflicts of interest. This will ensure that the peer review is conducted fairly and without bias. For more information regarding the program and how to apply to become a Volunteer Reviewer, please visit https://www.mdpi.com/reviewers.

## 4. The Review Process: From Submission to Final Decision

Once authors have familiarized themselves with the journal’s performance and open science practices, as well as read the detailed instructions provided on the journal’s website, the next step is the submission process itself. To help authors better understand how their work is evaluated, I would like to explain this process from the editor’s perspective, step by step.

### 4.1. Initial Quality Check

Upon submission of a new manuscript to the journal *Life*, the journal staff and in-house editorial team conduct an initial assessment to ensure that the manuscript adheres to editorial policies, ethical standards, financial disclosures, and data availability. This process usually takes 24 h. If any of these issues are identified, the manuscript is returned to the authors for changes or clarifications, or even rejection if one of these issues is found to be critical (e.g., plagiarism). If plagiarism is detected, the manuscript is immediately rejected. It is particularly crucial that this topic is highlighted because plagiarism has been the nightmare of publishers for decades. Even now, thousands of papers are retracted yearly due to plagiarism and scientific misconduct. At MDPI, this issue is not an issue because the publisher has strict ethical policies and standards in place in order to prevent plagiarism, data fabrication, and image manipulation in its publications. All MDPI submissions are checked for plagiarism using the industry-standard software iThenticate (Turnitin, Oakland, CA, USA). Such a strict technical check gives us, the academic editors, assurance that any potential misconduct has been minimized and that we may only worry about the scientific aspects of the submitted manuscripts.

### 4.2. Pre-Check Evaluation

After the initial quality check, our editorial office assigns the manuscript to an academic editor with relevant expertise for a pre-check evaluation of the proposal. The academic editor is usually a member of *Life*’s Editorial Board, but occasionally a Guest Editor is invited to serve instead. The editor provides a quick assessment of the manuscript based on scientific criteria, including, for example, whether the following are evident:

- The experiments, statistics, and other analyses are conducted at a high technical standard and are described in sufficient detail. Methods and reagents must be described in sufficient detail for another researcher to reproduce the experiments described. As clearly highlighted in our scope (https://www.mdpi.com/journal/life/about), our aim is to encourage scientists to publish their experimental and theoretical methods in as much detail as possible.
- Results are concise and clearly presented in figures and tables.
- Conclusions are presented appropriately and are supported by the provided data.
- The manuscript demonstrates a clear and coherent presentation, adhering to the standards of standard English usage. If the language used in a manuscript is difficult to comprehend or contains numerous errors, it is advised that the authors seek independent editorial assistance before submitting a revised version. This step is recommended to ensure that the manuscript is more easily understood, to expedite the review process, and to minimize delays in the publication of the research.

It is worth noting that we do not assess the quality of a manuscript based on biased perspectives, like the potential “impact” the paper may have. Many papers are unjustly rejected for being well-designed but lacking novel or appealing information, which, in my opinion, is highly unethical because it introduces subjective judgment and potential distortions into the evaluation process. By prioritizing impact over essential aspects of the scientific method, such as a rigorous methodology, appropriate research design, as well as the validity and reliability of the findings, biased decisions can undermine the integrity and objectivity of the peer review process. Assessing the worth of a manuscript should focus on crucial scientific factors and the execution of the research, rather than being influenced by subjective notions of impact or by particular interests. I highlighted this issue in particular nearly 10 years ago in my first letter as the new Editor-in-Chief of *Life*, quoting myself:

“*Instead of trying to predict if a theoretical study will be proven right or wrong or trying to predict the future impact of an experimental study, our focus on reviewing papers for consideration and possible publication will be on determining if the work is scientifically well written and presents coherent arguments. In 1974, Francis Crick, reflecting on the impact of the publication of his work on the structure of DNA that won him the Nobel Prize, suggested that it would be for historians to decide the impact of his work*”.

[9]

At this stage, we also have access to a list of suggested reviewers provided by either the authors or the assistant in-house editors. As such, we may check each suggestion, accepting or rejecting them, and suggest the names of additional qualified experts. In the end, we may choose to perform the following:

- Continue to peer review
- Send the manuscript back to the authors for revision
- Reject the manuscript

If the academic editors choose to continue the process, the assistant in-house editor invites the selected reviewers to provide feedback on the manuscript. After agreeing to review the manuscript, the external peer reviewers typically have 14 days to submit their review. Of course, reviewers may request any additional time if necessary. The journal office follows up with late reviewers and keeps the authors informed if there is any delay.

### 4.3. Review Reports

Reviewers are asked to evaluate the quality of the manuscript and to provide a recommendation to the academic editor as to whether a manuscript should be accepted, requires minor or major revisions, or should be rejected. We also ask reviewers to declare any potential conflicts and read the guidelines for reviewers [10].

General questions to help guide the writing of the review report for research articles include the following:

- Is the manuscript clear, relevant to the field and presented in a well-structured manner?
- Are the cited references mostly recent publications (within the last 5 years) and relevant? Does it include an excessive number of self-citations?
- Is the manuscript scientifically sound and is the experimental design appropriate to test the hypothesis?
- Are the manuscript’s results reproducible based on the details given in the methods section?
- Are the figures/tables/images/schemes appropriate? Do they properly represent the data? Are they easy to interpret and understand? Are the data interpreted appropriately and consistently throughout the manuscript? Please include details regarding the statistical analysis or data acquired from specific databases.
- Are the conclusions consistent with the evidence and arguments presented?

General questions to help guide the writing of the review report for review articles include the following:

- Is the review clear, comprehensive, and of relevance to the field? Is a gap in the knowledge identified?
- Was a similar review published recently and, if yes, is this current review still relevant and of interest to the scientific community?
- Are the cited references mostly recent publications (within the last 5 years) and relevant? Are any relevant citations omitted? Does it include an excessive number of self-citations?
- Are the statements and conclusions drawn coherently and supported by the listed citations?
- Are the figures/tables/images/schemes appropriate? Do they properly show the data? Are they easy to interpret and understand?

#### 4.3.1. Rating the Manuscript

During the manuscript evaluation, reviewers are also asked to rate the following aspects:

- Novelty: Is the question original and well-defined? Do the results provide an advancement in the current knowledge?
- Scope: Does the work fit the journal scope*?
- Significance: Are the results interpreted appropriately? Are they significant? Are all conclusions justified and supported by the results? Are the hypotheses carefully identified as such?
- Quality: Is the article written in an appropriate way? Are the data and analyses presented appropriately? Are the highest standards employed for the presentation of the results?
- Scientific Soundness: Is the study correctly designed and technically sound? Are the analyses performed to the highest technical standards? Are the data robust enough to draw conclusions? Are the methods, tools, software, and reagents described in sufficient detail to allow another researcher to reproduce the results? Are the raw data available and correct (where applicable)?
- Interest to the Readers: Are the conclusions interesting for the readership of the journal? Will the paper attract a wide readership, or be of interest only to a limited number of people? (Please see the Aims and Scope of the journal.)
- Overall Merit: Is there an overall benefit to publishing this work? Does the work advance the current knowledge? Do the authors address an important long-standing question with smart experiments? Do the authors present a negative result of a valid scientific hypothesis?
- English Level: Is the English language appropriate and understandable?

It is worth noting that MDPI allows reviewers to deposit their review activities into an ORCID iD if the reviewer’s ORCID account is connected to their MDPI Submission System (SuSy) account. To do this, reviewers should register for a SuSy account and connect their ORCID. Once the accounts are connected, reviewers can deposit their review records manually.

#### 4.3.2. Overall Recommendation

At the end of the report, reviewers are asked to provide an overall recommendation for the next processing stage of the manuscript, as follows:

- Accept in Present Form: The paper can be accepted without any further changes.
- Accept after Minor Revisions: The paper can, in principle, be accepted after revision based on the reviewer’s comments. Authors are given five days to make minor revisions.
- Reconsider after Major Revisions: The acceptance of the manuscript would depend on the revisions. The author needs to provide a point-by-point response or provide a rebuttal if some of the reviewer’s comments cannot be revised. A maximum of two rounds of major revision per manuscript is normally provided. Authors will be asked to resubmit the revised paper within ten days and the revised version will be returned to the reviewer for further comments. If the required revision time is estimated to be longer than 2 months, we will recommend that the authors withdraw their manuscript before resubmitting, so as to avoid unnecessary time pressure and to ensure that all manuscripts are sufficiently revised.
- Reject: The article has serious flaws, makes no original contribution, and the paper may be rejected with no offer of resubmission to the journal.

Note that the reviewer’s recommendation is visible only to the academic editor, not to the authors. In addition, reviewers should consider that the other reviewers evaluating a specific paper may possess different technical expertise and viewpoints. Consequently, the academic editor may need to make a decision based on conflicting advice. Therefore, the most valuable reports provide the academic editor with the information required in order to inform a decision. Presenting arguments both for and against publication is often more beneficial to editors than a direct recommendation in either direction.

At the end of the process, the academic editor grades the peer review reports from a scientific perspective and with regard to their general applicability to the improvement of the manuscript. The overall grading results are used as a reference for future invitations. Reviewers with high grades are invited more often and reviewers with low grades are eventually removed from the database.

### 4.4. Editorial Decision

Once all reviewers submit their reports, the academic editor receives a notification to log in to the system and make their final decision on each manuscript. The academic editor considers the feedback from the reviewers and their own evaluation of the manuscript in order to make a decision:

- Accept in present form
- Accept after minor revision
- Reconsider after major revision
- Reject and encourage resubmission
- Reject and decline resubmission

Decisions regarding the manuscript are conveyed to the corresponding author via an official letter, which is also shared with all co-authors. This letter includes the editorial decision with notes from the academic editor, the feedback from reviewers, as well as any additional requirements specified by the journal office. It is crucial for the authors to comprehend the implications of the rejection decisions. If the conclusions drawn in the manuscript require further support from additional experiments, the paper will be rejected, but the authors will be encouraged to resubmit it after conducting the necessary experiments. However, if the manuscript contains significant flaws, it will be rejected outright without the possibility of resubmission.

Making editorial decisions does not rely on vote counting or numerical rankings, and we do not always follow the recommendation of the majority. Our approach involves evaluating the strength of the arguments presented by each reviewer and the authors. Additionally, we may consider other relevant information that is not accessible to either party. Our primary responsibilities lie with the scientific community. Therefore, in determining how to best serve it, we carefully assess the merits of each paper in relation to the many others being considered.

When reviewers express conflicting opinions or when authors feel that they have been misunderstood regarding factual points, we may seek further guidance from the reviewers. It is crucial that reviewers are willing to provide additional advice upon request. However, we understand that reviewers are usually hesitant to engage in prolonged disputes. Therefore, we strive to minimize consultations to ensure that the authors receive a fair assessment.

### 4.5. Acceptance Decision

*Life* operates using two levels of acceptance decision. Once the scientific aspects of a manuscript are deemed satisfactory by the academic editor, an editorial acceptance decision is issued. This acceptance is provisional and subject to final checks for formatting and technical requirements. After fulfilling the final requirements, the journal office will send a formal acceptance decision to the authors. The manuscript will then proceed to production, undergoing professional copy-editing, English editing, proofreading by the authors, final corrections, pagination, and eventual publication.

### 4.6. Author Appeals

Authors may appeal a rejection by sending an e-mail to the Editorial Office of *Life*. The appeal must provide a detailed justification, including point-by-point responses to the reviewers’ and/or Editor’s comments using an appeal form. Appeals can only be submitted following a “reject and decline resubmission” decision, and should be submitted within three months of the decision date. Failure to meet these criteria will result in the appeal not being considered further. The Managing Editor will forward the manuscript and related information (including the identities of the referees) to a designated Editorial Board Member. The Academic Editor being consulted will be asked to provide an advisory recommendation on the manuscript and may recommend acceptance, further peer review, or uphold the original rejection decision. This decision will then be validated by the Editor-in-Chief. A rejection decision at this stage is final and cannot be reversed.

### 4.7. Open Peer Review

Authors are given the option for all review reports and editorial decisions to be published alongside their manuscript. In addition, reviewers can sign their review, i.e., identify themselves in the published review reports. Authors can alter their choice regarding open review at any time before publication. We encourage authors to take advantage of this opportunity as proof of the rigorous process employed in publishing their research. To guarantee impartial refereeing, the names of the referees will be revealed only if the referees agree to be named, and after a paper has been accepted for publication.

I am proud of being responsible for first implementing the open peer review system nearly 10 years ago in *Life* [11]. The first paper published under this new policy was a manuscript written by a Nobelist that was reviewed by three experts in the field [12]. The review reports were published as supplementary material to the review. This practice soon proved a popular option, and the initiative was extended to fourteen journals. By 2018, the option of Open Peer Review for submitted papers was available across the whole MDPI portfolio [13]. In 2020, MDPI published a total of 34,293 articles in Open Peer Review, which accounted for 21% of the total number of articles published in that year [14].

## 5. Final Remarks

As discussed in this editorial, *Life* is committed to providing a transparent, fair, and reliable peer review process. This is accomplished via a range of innovative features that have been refined over the past decade through close collaboration between *Life’s* board members and editorial staff, in conjunction with MDPI. Importantly, the publisher has consistently respected our autonomy in the decision-making process. Except for the Initial Quality Check, all decisions related to a submitted article are handled exclusively by the academic editors and board members. This foundational principle has underpinned the evaluation of over 12,500 articles submitted to the journal thus far. I can personally attest to this, as I have been associated with the journal since its launch in 2011; I was initially involved as a board member, and later as Editor-in-Chief. Furthermore, many esteemed colleagues whom I invited to join the *Life* board during its early days remain actively engaged, which ratifies our commitment to a reliable peer review process. With them, I have worked hard over the last decade to reach such high standards. As long as our autonomy in the decision-making process remains intact, our presence is an assurance of *Life’s* integrity.

The various strategies adopted by MDPI journals in their development also exemplify the autonomy wielded by the board members when making decisions. A noteworthy illustration is the conception of the open peer review system; when I idealized the implementation of this transparent system, only *Life* took the step forward. Subsequently, a few other journals embraced this approach, until the system was available across the MDPI portfolio. The decision to incorporate or eschew open peer reviews rested exclusively with the board members overseeing each journal’s operations. This independence exhibited by MDPI journals underscores the publisher’ commitment to fostering rigorous scholarly discourse and upholding the highest standards of academic publishing.

While the in-house assistant editors handle the day-to-day administrative tasks essential for journal operations, the board members are solely responsible for the scientific aspects of evaluating submitted manuscripts. This symbiotic relationship has been pivotal to our success. I hope that this editorial provides authors and readers with a deeper understanding of our values and unwavering dedication to our work, and in turn helps them to understand how their articles are being carefully evaluated.
